# Impact of the gut microbiome on skin fibrosis: a Mendelian randomization study

**DOI:** 10.3389/fmed.2024.1380938

**Published:** 2024-04-17

**Authors:** Zirui Zhao, Zhongye Xu, Dongming Lv, Yanchao Rong, Zhicheng Hu, Rong Yin, Yunxian Dong, Xiaoling Cao, Bing Tang

**Affiliations:** ^1^Department of Burn and Plastic Surgery, First Affiliated Hospital of Sun Yat-sen University, Guangzhou, China; ^2^Department of Dermatology, First Affiliated Hospital of Sun Yat-sen University, Guangzhou, China; ^3^Department of Plastic and Reconstructive Surgery, Guangdong Second Provincial General Hospital, Guangzhou, China

**Keywords:** gut microbiome, skin fibrosis, Mendelian randomization, gut-skin axis, single nucleotide polymorphisms

## Abstract

**Objective:**

Skin fibrosis is a lesion in the dermis causing to itching, pain, and psychological stress. The gut microbiome plays as an essential role in skin diseases developments. We conducted a Mendelian randomization study to determine the causal association between the gut microbiome and skin fibrosis.

**Methods:**

We retrieved valid instrumental variables from the genome-wide association study (GWAS) files of the gut microbiome (*n* = 18,340) conducted by the MiBioGen consortium. Skin fibrosis-associated data were downloaded from the GWAS Catalog. Subsequently, a two-sample Mendelian randomization (MR) analysis was performed to determine whether the gut microbiome was related to skin fibrosis. A reverse MR analysis was also performed on the bacterial traits which were causally associated with skin fibrosis in the forward MR analysis. In addition, we performed an MR-Pleiotropy Residual Sum and Outlier analysis to remove outliers and a sensitivity analysis to verify our results.

**Results:**

According to the inverse variance-weighted estimation, we identified that ten bacterial traits (*Class Actinobacteria, Class Bacteroidia*, *family Bifidobacteriaceae*, *family Rikenellaceae*, *genus Lachnospiraceae (UCG004 group)*, *genus Ruminococcaceae (UCG013 group)*, *order Bacteroidales*, *order Bifidobacteriales*, *genus Peptococcus* and *genus Victivallis*) were negatively correlated with skin fibrosis while five bacterial traits (*genus Olsenella*, *genus Oscillospira*, *genus Turicibacter*, *genus Lachnospiraceae (NK4A136group*), and *genus Sellimonas*) were positively correlated. No results were obtained from reverse MR analysis. No significant heterogeneity or horizontal pleiotropy was observed in MR analysis.

**Objective conclusion:**

There is a causal association between the gut microbiome and skin fibrosis, indicating the existence of a gut-skin axis. This provides a new breakthrough point for mechanistic and clinical studies of skin fibrosis.

## 1 Introduction

Skin fibrosis is an imbalance between extracellular matrix (ECM) synthesis and dermal degradation in the dermis. The remodelling process often leads to a loss of physiological architecture and skin malfunction including keloids, hypertrophic scars, systemic sclerosis, and dystrophic epidermolysis. Skin fibrosis is usually caused by surgery, burns and trauma and causes disfiguration, limitation of movement, or even significant psychological distress, such as keloids (1, 2). Keloids are common dermal skin fibrotic diseases. Owing to its aggressive proliferation, it is considered as benign skin tumour that harms the patient’s physiological and psychological health (3, 4). Keloids are refractory to cure, and their recurrence rate is over 45%. There are various treatment options, including intralesional steroid injections and surgical resection combined with postoperative radiation (4, 5). Therefore, it is important to explore the aetiology and pathogenic processes of skin fibrosis to provide novel insights into its treatment.

Many microbial communities reside in the intestine and affect human health and disease (6, 7). For example, the gut microbiome has been reported as a risk or preventive factor that can indirectly affect the response to immunotherapy in cancers (6, 8). The gut microbiome also influences the maintenance of maternal and foetal health during pregnancy (9). Recently, researchers have found that the gut microbiome plays a significant role in a wide variety of skin disorders, such as acne vulgaris, and reported the existence of a gut-skin axis (10, 11). However, the relationship between the gut microbiome and skin fibrosis remains unclear. Probing this association can be helpful for the treatment and clarification of the mechanism of skin fibrosis.

Unfortunately, the gut microbiome is associated with confounding factors, such as age, lifestyle, and living environment. Controlling these confounding biases inherent is difficult in the observational studies (12, 13). The advent of Mendelian randomization (MR) reduces the effect of these biases (14, 15). As Genome-wide association studies (GWAS) have greatly improved, we can observe the genetic susceptibility to the gut microbiome and skin fibrosis (16, 17). MR can be considered a new method for exploring the relationship between the gut microbiome and skin fibrosis (8, 13, 18).

In this study, we used two-sample MR analysis, an extension of the MR method, to analyse GWAS summary statistics from MiBioGen and the GWAS Catalog. Based on these results, we aimed to identify the role of the gut microbiome in skin fibrosis and propose new treatment strategies.

## 2 Materials and methods

### 2.1 Design of study

**Figure 1:**
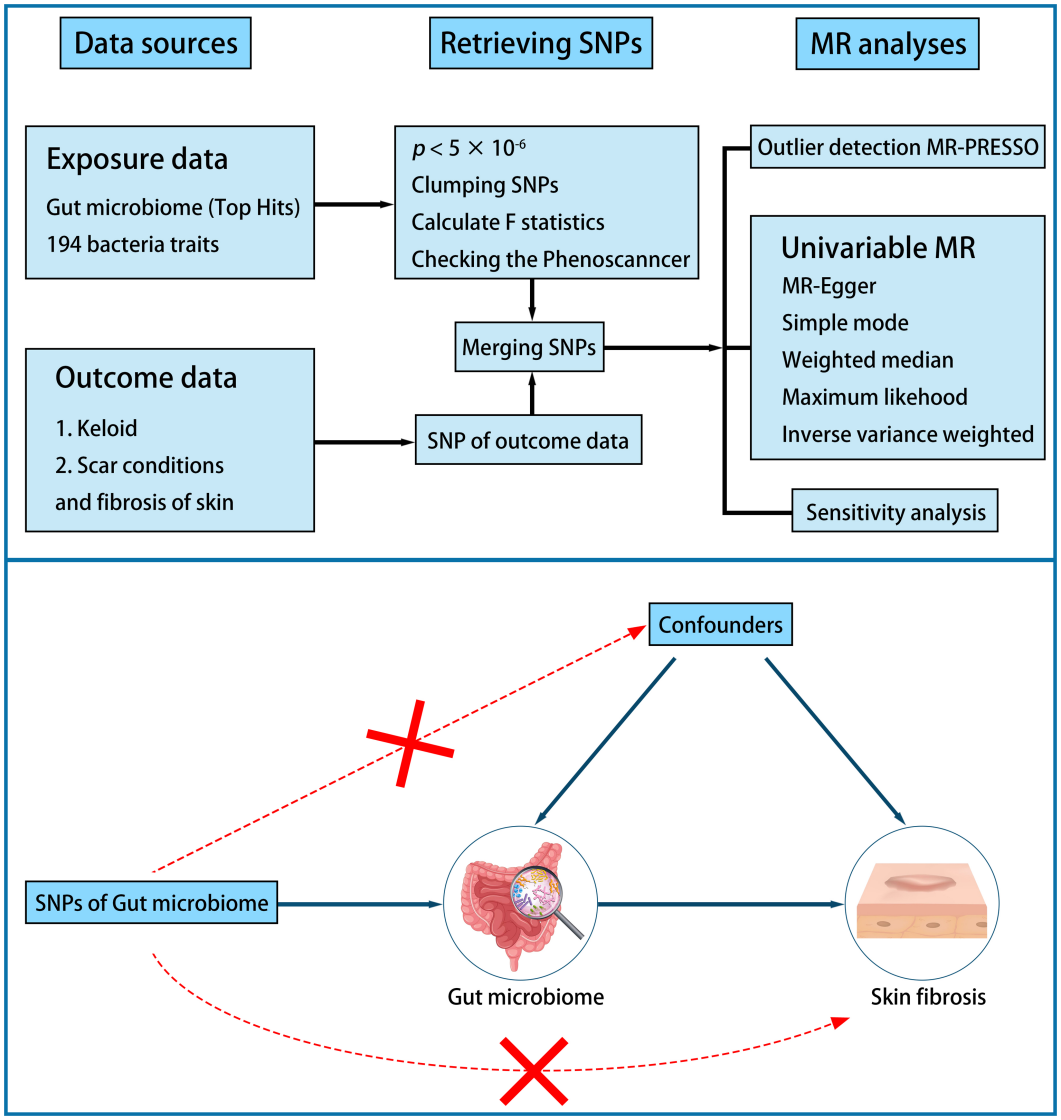
Flow plot and design of study.

A two-sample MR study was performed to examine the causal association between the gut microbiome and skin fibrosis (Figure 1). First, we identified a valid instrumental variable (IV). IVs must be associated with exposure but not with confounders. IVs must relate to outcomes only through exposure. Second, the appropriate IVs were subjected to MR analysis. Sensitivity analysis was conducted to verify these results (18, 19).

### 2.2 Data preparation

Human gut microbiome GWAS statistics were retrieved from the MiBioGen Consortium, an international project on genome-wide genotypes, and 16S faecal microbiome data (17, 20). In total, 194 bacterial traits were identified in the dataset. In this project, 18,340 individuals from 24 cohorts were included, of which 13,266 were of European ancestry. Thus, participants of European ancestry were selected to reduce the influence of ethnicity. We obtained GWAS data for keloids from GCST90044522. This study included 201 cases and 456,147 controls of European descent. In addition, summary genetic data for scar conditions and skin fibrosis were downloaded from GCST90044521 including 1,887 cases and 454,461 controls (Table 1) (21). The detail of gut microbiome was also shown (Supplementary Table S1).

**Table 1:** Overview of study data.

Study name	Year	Trait	Source	Sample size	Number of SNPs	Population
MiBioGen	2023	Gut microbiome	MiBioGen consortium	18,340	121,548	United Kingdom, Finland, Sweden, etc.
GCST90044522	2021	Keloid scar	GWAS Catalog	456,348	11,842,647	European (United Kingdom)
GCST90044521	2021	Scar conditions and fibrosis of skin	GWAS Catalog	456,348	11,842,647	European (United Kingdom)

### 2.3 Single nucleotide polymorphisms selection

We selected appropriate SNPs as IVs to reduce biases and improve the credibility of the results. The selection criteria were as follows: (1) the SNP was associated with the gut microbiome (*p* < 5 × 10^−6^); (2) the SNP was independent (*r*^2^ < 0.01 and clump distance >10,000 kb, EUR population reference); (3) it was highly correlated to exposure traits (the *F*-statistic >10); (4) palindromic SNPs were harmonized in exposure and outcome data; and (5) the SNPs associated with potential risk factors, such as inflammatory factor, were deleted based on PhenoScanner V2 (5, 16, 18, 22, 23).

### 2.4 Statistical analyses

Before the MR analysis, we performed an MR-Pleiotropy Residual Sum and Outlier (MR-PRESSO) analysis to identify and remove outliers.

We performed MR analysis using the inverse variance-weighted (IVW), MR–Egger, weighted median, simple mode, and weighted mode methods. We chose the IVW as the primary method because it can provide more robust estimates in a broader set of scenarios. Other methods serve as complementary methods (8, 18, 24).

To verify our study, we performed a sensitivity analysis using Cochran’s test. Heterogeneity was tested using Cochran’s *Q* test. If the *Q* statistic is *p* < 5 × 10^−2^, it indicates the presence of heterogeneity (25, 26). We tested for the pleiotropic effect using the MR–Egger regression intercept. There was no horizontal pleiotropy of the study when *p* > 5 × 10^−2^ (24). Furthermore, a leave-one-out analysis was used to assess the horizontal pleiotropy. Finally, reverse MR analysis was performed using the same methods and settings (8, 13).

Statistical analyses were performed using R version 4.3.0, and the MR analysis was performed using the TwosampleMR package (version 0.5.7) (27).

## 3 Results

### 3.1 IVs associated with the gut microbiome

1,384 SNPs were retrieved from 194 bacterial traits, including phyla, classes, orders, families, and genus levels by the genera. There was no bias in weak IVs (all *F* > 17). Additional details are provided in Supplementary Table S2.

### 3.2 Causal effects of the gut microbiome on skin fibrosis

**Figure 2:**
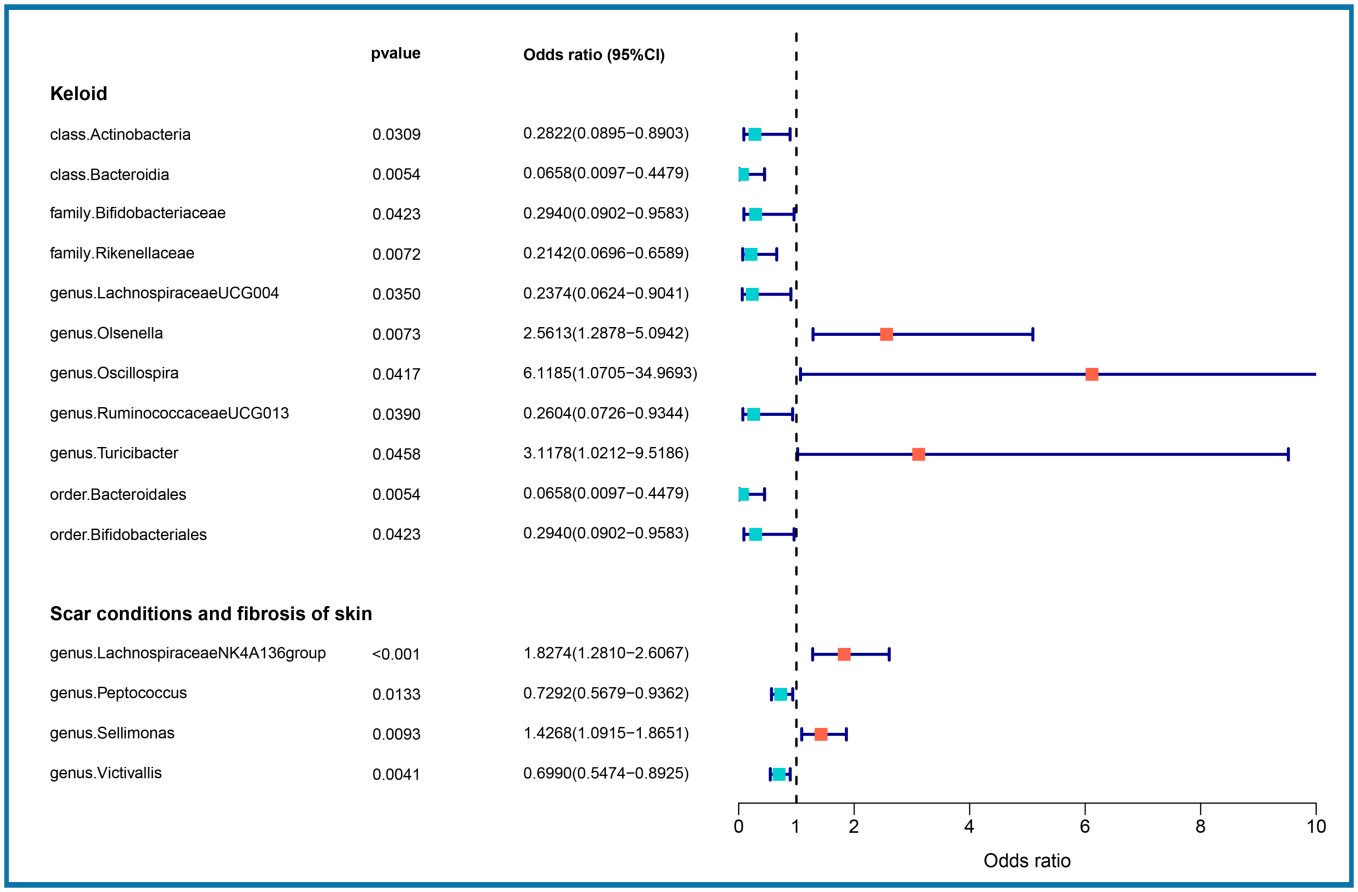
Association between gut microbiome (15 bacterial features of MiBioGen consortium) and skin fibrosis (GCST90044521 & GCST90044522) (*p* < 5 × 10^−6^). Color code = “#48D1CC,” “#FA8072” and “#FF0000.”

**Figure 3:**
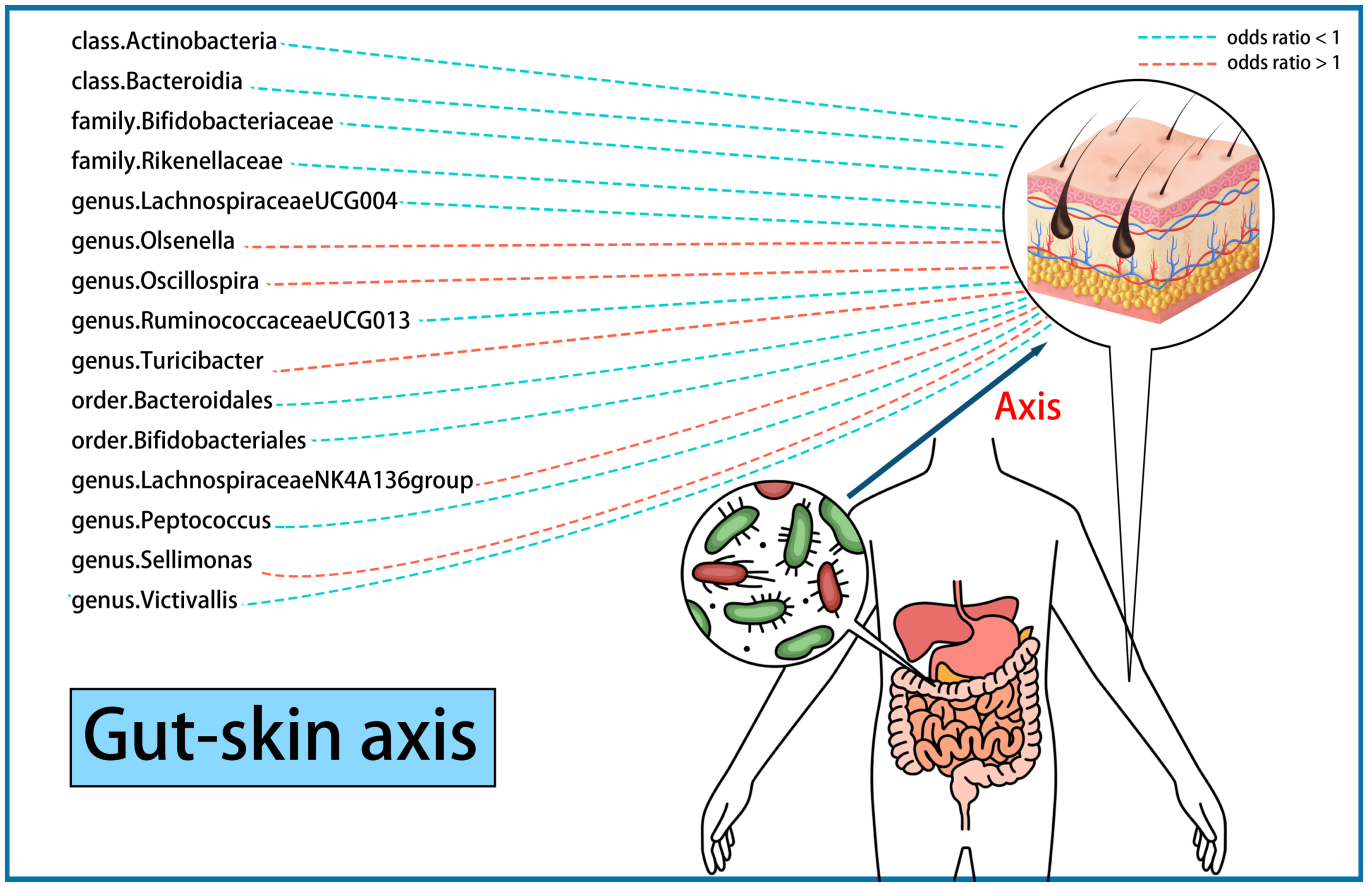
Existence of gut-skin axis supported by a two-sample Mendelian randomization (15 bacterial features of MiBioGen consortium). Color code = “#48D1CC,” “#FA8072” and “#FF0000.”

We performed an MR analysis of 194 bacterial traits (Supplementary Table S3). Most of these factors were not associated with skin fibrosis. However, 15 bacterial features were potentially associated with skin fibrosis. The odds ratio (OR) of *Class Actinobacteria* was 0.2822, while the 95% confidence interval (CI) was 0.0895–0.8903, and the *p* value was 3.09 × 10^−2^. The OR of *Class Bacteroidia* was 0.0659, the 95% CI was 0.0097–0.4479, and the *p* value was 5.4 × 10^−3^. The OR of *family Bifidobacteriaceae* was 0.2940, while the 95% CI was 0.0902–0.9583, and the *p* value was 4.23 × 10^−2^. The OR of *family Rikenellaceae* was 0.2142, the 95% CI was 0.0697–0.6590 and the *p* value was 7.2 × 10^−3^. The OR of *genus Lachnospiraceae (UCG004 group)* was 0.2374, the 95% CI was 0.0624–0.9041 and the *p* value was 3.5 × 10^−2^. The OR of *genus Olsenella* was 2.5613, the 95% CI was 1.2878–5.0942 and the p value was 7.3 × 10^−3^. The OR of *genus Oscillospira* was 6.1185, the 95% CI was 1.0705–34.9693 and the *p* value was 4.17 × 10^−2^. The OR of *genus Ruminococcaceae (UCG013 group)* was 0.2604, while the 95% CI was 0.0726–0.9344 and the *p* value was 3.9 × 10^−2^. The OR of *genus Turicibacter* was 3.1178, while the 95% CI was 1.0212–9.5186 and the p value was 4.59 × 10^−2^. The OR of *order Bacteroidales* was 0.0658, while the 95% CI was 0.0097–0.4479 and the *p* value was 5.4 × 10^−3^. The OR of *order Bifidobacteriales* was 0.2940, while the 95% CI was 0.0902–0.9583 and the *p* value was 4.23 × 10^−2^. These were causally associated with keloids. Moreover, the OR of *genus Lachnospiraceae (NK4A136group)* was 1.8274, while the 95% CI was 1.2810–2.6067 and the *p* value was 9 × 10^−4^. The OR of *genus Peptococcus* was 0.7292, while the 95% CI was 0.5679–0.9362 and the p value was 1.33 × 10^−2^. The OR of *genus Sellimonas* was 1.4268, the 95% CI was 1.0915–1.8651 and the *p* value was 9.3 × 10^−3^. The OR of *genus Victivallis* was 0.6990, while the 95% CI was 0.5474–0.8925 and the *p* value was 4.1 × 10^−3^. The OR > 1 means that the gut microbiome was positively associated with skin fibrosis while the OR < 1 means negatively associated with skin fibrosis. Besides, *p* < 0.05 means the result is statistically significant (Figure 2). These results indicate that the gut can affect skin disease, supporting the existence of the gut-skin axis (Figure 3). Refer to single nucleotide polymorphisms annotator (SNIPA), the genes associated with SNP of these bacterial trait were exhibited in Supplementary Table S4.

In addition, we used the traditional GWAS significance threshold (*p* < 5 × 10^−8^) as the selection criterion for the IVs. However few SNPs remained, and no significant results were observed in Supplementary Table S5.

In the reverse MR analysis, we found that there were not enough SNPs that were limited by the size of the gut microbiome. Using the selection criteria, no IVs were found in the skin fibrosis and gut microbiome data simultaneously.

### 3.3 Sensitivity analysis

According to Cochran’s *Q* test and MR–Egger regression intercept analysis, there was no significant heterogeneity or directional horizontal pleiotropy. Furthermore, the results of MR-PRESSO showed no outliers (Table 2). Leave-one-out analysis was performed to verify the MR results. Forest plots, scatter plots and funnel plots for the causal association between the gut microbiome and skin fibrosis are also shown (Figure 4, Supplementary Figures S1–S14).

**Table 2:** MR results of causal relationship between gut microbiome and skin fibrosis.

Gut microbiome	Method	OR (95% CI)	*p*-value	*Q* statistic	*Q p*-value	Egger intercept	Intercept *p*-value	MR-PRESSO
Keloid								
*class.Actinobacteria*	IVW	0.2822 (0.0895–0.8903)	0.0309	3.9086	0.7902	0.1394	0.4067	—
*class.Bacteroidia*	IVW	0.0659 (0.0097–0.4479)	0.0054	6.6544	0.2476	0.1818	0.3859	—
*family.Bifidobacteriaceae*	IVW	0.2940 (0.0902–0.9583)	0.0423	2.1649	0.9040	0.0789	0.6206	—
*family.Rikenellaceae*	IVW	0.2142 (0.0697–0.6590)	0.0072	6.9211	0.6453	−0.0421	0.7085	—
*genus.LachnospiraceaeUCG004*	IVW	0.2374 (0.0624–0.9041)	0.0350	1.1241	0.9805	−0.0233	0.9238	—
*genus.Olsenella*	IVW	2.5613 (1.2878–5.0942)	0.0073	2.9761	0.8118	0.1516	0.4188	—
*genus.Oscillospira*	IVW	6.1185 (1.0705–34.9693)	0.0417	0.3714	0.8305	−0.2023	0.7318	—
*genus.RuminococcaceaeUCG013*	IVW	0.2604 (0.0726–0.9344)	0.0390	6.3107	0.3893	−0.0934	0.5678	—
*genus.Turicibacter*	IVW	3.1178 (1.0212–9.5186)	0.0459	4.3163	0.5048	0.0031	0.9940	—
*order.Bacteroidales*	IVW	0.0658 (0.0097–0.4479)	0.0054	6.6544	0.2476	0.1818	0.3859	—
*order.Bifidobacteriales*	IVW	0.2940 (0.0902–0.9583)	0.0423	2.1649	0.9040	0.0789	0.6206	—
Scar conditions and fibrosis of skin								
*genus.LachnospiraceaeNK4A136group*	IVW	1.8274 (1.2810–2.6067)	0.0009	5.0928	0.5320	−0.0096	0.7544	—
*genus.Peptococcus*	IVW	0.7292 (0.5679–0.9362)	0.0133	3.4440	0.8411	−0.0091	0.8772	—
*genus.Sellimonas*	IVW	1.4268 (1.0915–1.8651)	0.0093	2.4355	0.4871	−0.0504	0.6215	—
*genus.Victivallis*	IVW	0.6990 (0.5474–0.8925)	0.0041	2.6004	0.6267	−0.1390	0.2982	—

*p*-value < 0.05 shows that there is a potential association between gut microbiome and skin fibrosis. *Q p*-value < 0.05 shows that there is heterogeneity in the analysis. Intercept *p*-value < 0.05 shows that there is pleiotropic effect in the analysis and the result is not credible.

**Figure 4:**
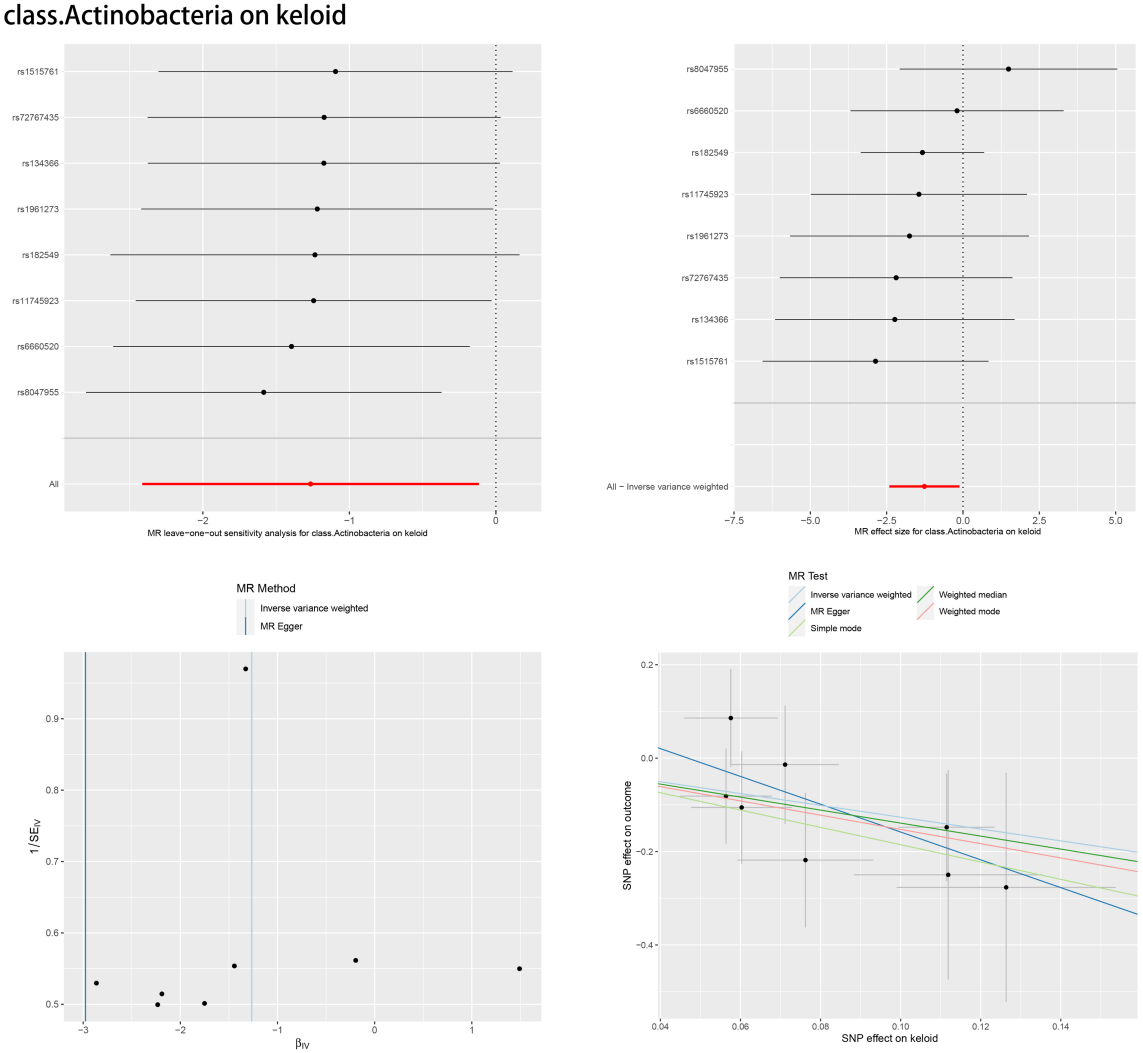
Leave-one-out plot, forest plot, funnel plot and scatter plot of *class. Actinobacteria*.

## 4 Discussion

In the occurrence of skin disease, researchers observe microbial dysbiosis both in the gut and skin (10, 28). For example, the microbiological composition of healthy skin is balanced with proper quantities of human and microbial antimicrobial peptides (AMPs). AMPs are a secretory product of epithelial and immune cells regulated by the Toll-like receptor (TLR) pathway. AMPs can maintain intestinal homeostasis and prevent the entry of gut bacteria into the bloodstream. When TLR pathways are inhibited, gut allostasis occurs. Dysbiosis of the gut microbiome influences the conversion of complex polysaccharides into vitamins and short-chain fatty acids (SCFAs) to improve the integrity of the gut barrier (10). They may also affect nitric oxide (NO) production and influence blood flow through the denitrification pathway (29). Impairment of intestinal epithelial cells leads to reduce production of AMPs and immunoglobulin A (IgA), which exacerbates gut microbial dysbiosis. As a result, gut bacteria enter the bloodstream through the gut wall and then reach the skin, causing dysbiosis of the skin microbiome and tissue inflammation (10, 11, 30).

The skin performs its functions and undergoes constant renewal during homeostasis. Gut microbial dysbiosis can cause skin allostasis (11, 31). For example, the dysbiosis of Firmicutes and Bacteroides alters the serological cytokine levels and promotes inflammation, leading to the development of acne vulgaris (31). This interaction is mainly mediated by the immune system (10, 32). Additionally, bacterial metabolites, such as butyrate, are related to the integrity of the epithelial barrier which engages to protect the skin (10, 30, 33, 34). Intact skin is crucial for maintaining homeostasis (35). This intricate interaction is known as the gut-skin axis. However, there are few reports on the role of gut-skin axis in skin fibrosis (28). Thus, we designed an MR analysis to determine the impact of the gut microbiome on the skin.

In this study, we evaluated the casual association between the abundance of specific bacterial signatures and the risk of skin fibrosis. Ten bacterial traits showed protective effects against skin fibrosis: *Class Actinobacteria, Class Bacteroidia, family Bifidobacteriaceae, family Rikenellaceae, genus Lachnospiraceae (UCG004 group), genus Ruminococcaceae (UCG013 group), order Bacteroidales, order Bifidobacteriales, genus Peptococcus, and genus Victivallis*. The *genus Olsenella, genus Oscillospira, genus Turicibacter*, *genus Lachnospiraceae (NK4A136group)*, and *genus Sellimonas* are risk factors for skin fibrosis. In a randomised clinical pilot trial, participants consecutively consumed milk containing *family Bifidobacteriaceae* twice a day for 8 weeks. Compared with the pre-intake period, researchers found that the relative abundance levels of *family Bifidobacteriaceae* were significantly increased, and the skin condition of participants improved (36). *Genus Lachnospiraceae (UCG004 group)* acts as probiotics and increases the production of SCFAs including butyric acid for skin homeostasis (33, 37). Sodium butyrate dampens the profibrotic response induced by TGF-β1 in human dermal fibroblasts (38). A study reported higher abundance levels of *genus Turicibacter* in patients with systemic lupus erythematosus than in healthy individuals (39). These results are consistent with those of our study and support the existence of a gut-skin axis. Besides, Order *Bacteroidales* and *genus Olsenella* are associated with checkpoint blockade immunotherapy in melanoma (40, 41). The mechanism underlying other bacterial traits in the gut-skin axis remains to be elucidated.

There are currently no effective treatments for skin fibrosis (2). With the development of research on gut microbiome and skin fibrosis, we can elucidate the mechanisms of skin fibrosis and explore new therapeutic targets. These include the intake of probiotics, transplantation of the faecal microbiome, dietary modification, and drug–microbiome combination treatment (42–45). Relative abundances of gut microbiome were regularly detected in the suspected population. Before the disease onset, early screening and diagnosis can be performed by engineering the gut microbiome and restoring intestinal homeostasis. In the context of skin fibrosis, we propose novel and effective therapeutic strategies based on changes in specific gut microbiome abundance levels. Based on these levels, we can evaluate the effects of treatment and adjust therapeutic strategies for precise treatment. After treatment, diet can be leveraged to optimise SCFA production, maintain a healthy gut-skin axis, and reduce the risk of recurrence (46). To achieve this goal, we not only need appropriate data analysis to probe the causal association between the gut microbiome and skin fibrosis but also require a large number of rigorous clinical trials for validation.

Our study has some limitations. First, the results might not be entirely applicable to individuals of non-European descent because almost all samples are European, and only a few gut microbiome samples are from other races (17, 20, 21). Second, there might be a possibility of identifying false-positive findings owing to the absence of no Bonferroni correction. However, the results with IVW-derived *p* values less than 0.05 should also be treated cautiously (14). Third, the GWAS data for the gut microbiome were coordinated using by 16S rRNA gene sequencing, and the lowest taxonomic level was the genus. As a result, it is difficult to estimate the relationships between specific strains or species and skin fibrosis (17, 18). Additionally, we cannot exclude a reverse causal association between the gut microbiome and skin fibrosis because of the relatively small size of the gut microbiome. We hope that future GWAS data of the gut microbiome will be more sufficient, and we can perform analyses between a specific species and skin fibrosis in both European and non-European populations to reduce bias and improve universality.

## 5 Conclusion

The MR analysis revealed a causal association between the gut microbiome and skin fibrosis, supporting the existence of the gut-skin axis. When confronted with skin fibrosis, we also need to be cautious of the condition of the gut microbiome. Abundance of the gut microbiome can be a potential biomarker for early diagnosis and treatment of skin fibrosis. The role of the gut-skin axis in skin fibrosis requires further investigation.

## Data availability statement

The datasets presented in this study can be found in online repositories. The names of the repository/repositories and accession number(s) can be found in the article/Supplementary material.

## Author contributions

ZZ: Conceptualization, Data curation, Formal analysis, Investigation, Methodology, Resources, Software, Validation, Visualization, Writing – original draft, Writing – review & editing. ZX: Conceptualization, Investigation, Software, Writing – original draft, Writing – review & editing. DL: Conceptualization, Investigation, Software, Writing – original draft, Writing – review & editing. YR: Writing – review & editing. ZH: Writing – review & editing. RY: Funding acquisition, Writing – review & editing. YD: Writing – review & editing. XC: Project administration, Supervision, Writing – review & editing. BT: Funding acquisition, Project administration, Supervision, Writing – review & editing.
